# The Association Between Dehydration and the Prognosis of Sudden Sensorineural Hearing Loss

**DOI:** 10.1097/ONO.0000000000000041

**Published:** 2023-10-10

**Authors:** Yasunori Abe, Masahiro Okada, Keiko Tanaka, Kensuke Toyama, Yoshito Miyamoto, Naohito Hato

**Affiliations:** 1 Department of Otolaryngology, Head and Neck Surgery, Ehime University School of Medicine, Toon, Japan; 2 Department of Otolaryngology, Jyuzen General Hospital, Niihama, Japan; 3 Department of Epidemiology and Public Health, Ehime University Graduate School of Medicine, Toon, Japan; 4 Integrated Medical and Agricultural School of Public Health, Ehime University, Matsuyama & Toon, Japan; 5 Department of Pharmacology, Ehime University Graduate School of Medicine, Toon, Japan.

**Keywords:** BUN/Cre ratio, Dehydration, Plasma osmolality, Prognosis, Sudden sensorineural hearing loss

## Abstract

**Background:**

There is an urgent need to identify undetermined risk factors for sudden sensorineural hearing loss (SSNHL) for the development of effective treatment strategies. SSNHL is likely associated with vascular insufficiency; however, no study has evaluated the relationship between dehydration and SSNHL.

**Objective:**

This study aimed to investigate the role of dehydration in the development and prognosis of sudden sensorineural hearing loss.

**Study Design:**

Retrospective case-control study.

**Setting:**

Secondary referral hospital.

**Patients and Interventions:**

This was a comparative study that compared dehydration parameters between healthy subjects without SSNHL (n = 94) and patients with SSNHL (n = 94). The study also evaluated the effect of dehydrated conditions on the prognosis of SSNHL.

**Main Outcome Measures:**

We compared dehydration parameters, such as the blood urea nitrogen-to-creatinine ratio (BUN/Cre) and plasma osmolality (Posm), between matched healthy subjects without SSNHL and patients with SSNHL. To evaluate the effect of dehydrated conditions on the SSNHL prognosis, the SSNHL patients were divided into 2 groups based on the cutoff value obtained from the receiver operating characteristic analysis: hydrated (n = 50; BUN/Cre <21.4) and dehydrated (n = 44; BUN/Cre ≥21.4) groups. Subsequently, the severity and prognosis of SSNHL were analyzed.

**Results:**

The dehydration parameters, BUN/Cre and Posm, were significantly higher in patients with SSNHL than in healthy subjects. The initial hearing levels and SSNHL grades were worse in the dehydrated group than in the hydrated group. Moreover, a dehydrated condition (BUN/Cre ≥21.4) was associated with a poor SSNHL prognosis in all models of the multiple logistic regression analysis.

**Conclusions:**

The dehydration parameters of BUN/Cre and Posm were higher in patients with SSNHL than in healthy subjects. Additionally, a dehydrated condition (BUN/Cre ≥21.4) was an independent prognostic factor for SSNHL. Level of evidence: Level 4.

Sudden sensorineural hearing loss (SSNHL) is an acute hearing loss disease characterized by a rapid hearing loss of more than 30 decibels in hearing level (dB HL) in 3 contiguous frequencies within 72 h (1). The estimated incidence rate of SSNHL in the United States is 11 to 77 per 100,000 people per year (2), while it is 60.9 per 100,000 population in Japan (3).

Various etiologies have been proposed for SSNHL, including vascular insufficiency (4), viral infection (5), autoimmune mechanisms (6), and stress responses (7). Although the pathogenesis of SSNHL remains unknown, circulatory disturbances are recognized as the most plausible causes (4,8). Lifestyle-related diseases that induce arteriosclerosis are associated with SSNHL prognosis (9) and patients with SSNHL have a higher risk for stroke than those without SSNHL (10).

Dehydration causes vascular insufficiency, has a negative impact on the cardiovascular system, and sometimes induces a fatal condition and disease (11). Cortés-Vicente et al. (12) showed that dehydration was associated with an unfavorable outcome of a stroke at discharge, and El-Sharkawy et al. (13) showed that dehydration induced acute kidney injury and increased the length of hospital stay and mortality. To the best of our knowledge, no previous clinical studies have examined the association between dehydration and SSNHL.

We hypothesized that dehydration negatively influences the prevalence, severity, and prognosis of SSNHL. We conducted this cohort study to investigate the relationship between SSNHL and dehydration status using Posm and BUN/Cre.

## MATERIALS AND METHODS

### Data Sources and Study Exclusion Criteria

This was a retrospective case–control study, including patients with SSNHL who were hospitalized at Jyuzen General Hospital from January 2012 to March 2022 and participants in health checkups at Jyuzen General Hospital Healthcare Center in 2021. SSNHL was defined as SSNHL of more than 30 dB HL in at least 3 contiguous frequencies within 72 h (1). The exclusion criteria were: patients with SSNHL whose treatment was initiated more than 2 weeks after disease onset; patients who, during treatment and follow-up periods, were diagnosed with other diseases related to hearing loss, such as vestibular schwannoma, Meniere’s disease, or perilymphatic fistula; patients with hearing impairments in the contralateral ear due to SSNHL, other causes, and age-related hearing loss, and so on, since we could not distinguish whether their hearing levels had recovered completely or not; and patients with missing clinical and/or biochemical data. “Complete recovery” was defined as the restoration of the pure-tone average (PTAv) on the impaired side to within 10 dB of the better side. Consequently, patients with hearing impairments in the contralateral ear were excluded.

In our hospital, treatment for SSNHL comprises intravenous systemic prednisolone, oral vitamin B_12_ (1500 μg/day), and oral adenosine triphosphate (300 mg per day). Prednisolone is tapered gradually over 3 weeks from 60 mg per day. Additional treatments included intratympanic dexamethasone injection (IT-DEX) and prostaglandin I2 (PGI2) for patients nonresponsive to systemic prednisolone. IT-DEX was administered to hospitalized patients concurrently with intravenous systemic prednisolone, and intravenous PGI2 was administered at a dosage of 5 μg/day.

This study was approved by the Ethics Committee of the Ehime University Graduate School of Medicine (Ehime, Japan) (Approval number: 2211015). Informed consent in this study was obtained through opt-out.

### Audiological Examination

Hearing tests were performed at the initial visit and every week from the start of treatment. Hearing outcomes were assessed 1 month after treatment. When complete recovery was achieved during treatment, we defined the hearing level as the final result after confirming the absence of re-aggravation. Patients with SSNHL were followed up until their hearing levels no longer changed.

Audiometric hearing assessments were performed in the form of air and bone conduction threshold testing using a pure-tone audiometer (AA-76S; Rion, Tokyo, Japan) in a soundproof booth. Air conduction thresholds were obtained from 0.125 to 8 kHz, and bone conduction thresholds were obtained from 0.25 to 4 kHz using a manual testing protocol. Hearing levels were determined using air conduction hearing levels. PTAv was calculated at the 4-frequency average of 500, 1000, 2000, and 4000 Hz.

As defined by the Ministry of Health and Welfare in Japan, the grade of hearing loss and recovery rate (15) were obtained from audiogram data. Patients with grades 1 and 2 hearing loss (PTAv <60 dB) were categorized as mild hearing loss, and those with grades 3 and 4 (PTAv ≥60 dB) were categorized as severe hearing loss.

### Demographics

Among patients with SSNHL, information regarding risk factors of cardiovascular diseases, including smoking habits, hypertension, dyslipidemia, diabetes, and current use of antihypertensive, antidyslipidemic, and antidiabetic agents was collected from medical records. Among the health checkup participants, self-administered questionnaires were obtained concerning this information.

### Laboratory-based Characteristics

Blood samples were collected before treatment. Complete blood counts were performed using an XE-5000 (Sysmex Corporation, Japan), blood biochemical examinations were performed using JCA-BM6050 (Japan Electron Optics Laboratory, Japan), and blood glucose levels were analyzed using GA-1170 (ARKRAY, Inc., Japan).

Posm was calculated as follows: Posm (mOsm/L) = 2 × plasma sodium + BUN/2.8 + blood glucose/18.

### Statistical Analysis

The Shapiro–Wilk test was used to determine whether the samples were normally distributed. Depending on the distribution of data, the Student *t* test or Wilcoxon test was used to analyze the differences in the values of the variables between the groups. *χ*^2^ tests were performed to find the significant difference in categorized variables between the groups. Receiver operating characteristic (ROC) curves were used to examine the predictive value of recovery rates, and the cutoff points were selected as the one that minimizes the Euclidean distance between the ROC curve and the point (0, 1). ROC tests were performed to compare the areas under the curve (AUC). Univariate and multivariate logistic regression analyses were used to assess baseline parameters, risk factors, and medications associated with SSNHL prognosis. To match the population of patients with SSNHL and healthy subjects, a one-to-one nearest matching analysis based on the estimated propensity scores of each subject was performed (adjusted for age, sex, and systolic blood pressure).

The data were expressed as means ± standard deviations, or medians and interquartile ranges, or n (%). The results of the single regression and logistic regression analyses were expressed as odds ratios (OR) with 95% confidence intervals (CI). Statistical significance was defined as a *P* value of less than 0.05. All statistical analyses were performed using R version 4.2.2 (The R Foundation for Statistical Computing, Austria).

## RESULTS

### Hydration Status in Patients With SSNHL Compared With the Healthy Population

Supplemental Figure 1, http://links.lww.com/ONO/A18 shows the schema of the first cohort used to compare patients with SSNHL with the healthy population. As shown on the left side of this schema, 109 patients with SSNHL were enrolled; and 7 patients with hearing impairment in the contralateral ear and 8 patients with missing biochemical data were excluded. Ultimately, 94 eligible patients with SSNHL were included in this study. As shown on the right side of the schema, 1165 participants in the medical checkup were enrolled as the control group, and 13 participants with missing biochemical data were excluded. Matched with the SSNHL-eligible patients (adjusted for age, sex, and systolic blood pressure), 94 subjects were selected as the control group.

A comparison of the characteristics between the 2 groups is shown in Supplemental Table 1, http://links.lww.com/ONO/A22. Because the 2 groups were matched, no parameters differed significantly between the groups. As shown in Supplemental Table 2, http://links.lww.com/ONO/A23, multiple laboratory-based data significantly differed between the groups, whereas the median of all variables remained within normal ranges.

As shown in Supplemental Figure 2, http://links.lww.com/ONO/A19, BUN/Cre was 21.1 (17.7–25.4) in patients with SSNHL and 18.8 (15.3–22.6) in the control; BUN/Cre was significantly higher in patients with SSNHL compared with the control (*P* = 0.001, effect size = 0.867). Posm was 300.6 ± 5.0 mOsm/L in patients with SSNHL and 292.2 ± 4.1 mOsm/L in the control; it was significantly higher in patients with SSNHL than that in the control (*P* < 0.001, effect size = 1.832).

### ROC Analysis for Hydration Status in Patients with SSNHL

Supplemental Figure 3, http://links.lww.com/ONO/A20 shows the AUCs of the dehydration parameters for the prognosis of patients with SSNHL: BUN/Cre 0.66 (95% CI 0.55–0.77), Posm 0.56 (95% CI 0.44–0.68). ROC tests revealed that there was no significant difference between BUN/Cre and Posm (*P* = 0.25), whereas the AUC of BUN/Cre was higher than that of Posm. For the next analysis, the cutoff point of BUN/Cre was selected as 21.4 with a sensitivity of 73% and a selectivity of 62% (Supplemental Figure 3, http://links.lww.com/ONO/A20).

### Association Between Dehydration and Severity and Prognosis of SSNHL

**FIG 1.**
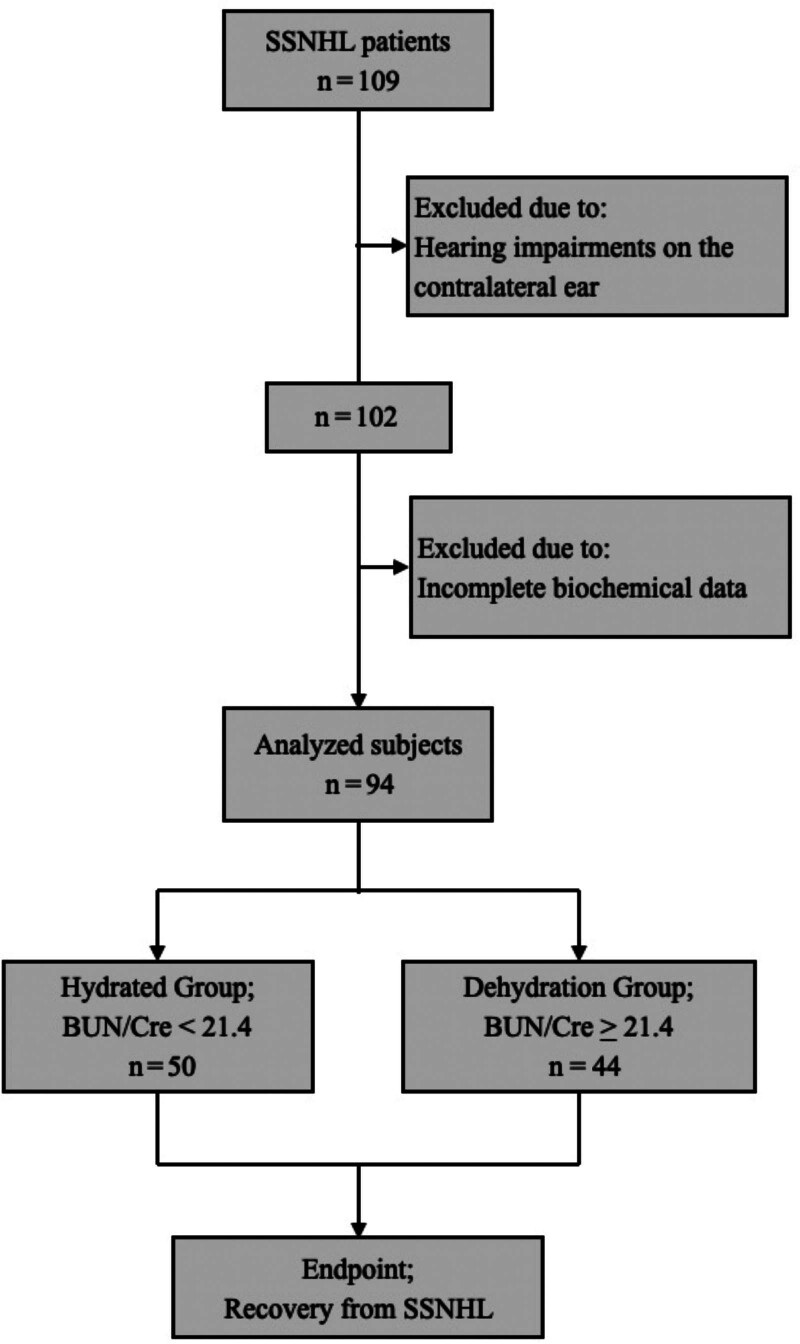
Study flow diagram to evaluate dehydration for the prognosis of SSNHL. To evaluate the relationship between dehydration and the prognosis of SSNHL, patients with SSNHL were divided into two groups. BUN/Cre indicates blood urea nitrogen-to-creatinine ratio; SSNHL, sudden sensorineural hearing loss.

To evaluate the effect of dehydration on SSNHL, the 94 eligible patients with SSNHL were divided using the cutoff point (BUN/Cre = 21.4) reported in the last paragraph into 2 groups: hydrated (BUN/Cre < 21.4) and dehydrated (BUN/Cre ≥ 21.4) (Fig. 1). A comparison of the characteristics of the 2 groups is presented in Table 1. Age and sex differed significantly between the hydrated and dehydrated groups. Table 2 compares the clinical parameters of patients with SSNHL between the hydrated and dehydrated groups. Parameters affected by the body water balance, such as BUN, creatinine, Posm, and total cholesterol levels, were higher in the dehydrated group than in the hydrated group. The initial hearing levels and morbidity of severe hearing loss were higher in the dehydrated group than in the hydrated group (*P* = 0.03, effect size = 0.864; *P* = 0.049, effect size = 0.227, respectively).

**TABLE 1.** The comparison of baseline characteristics between patients with SSNHL in the hydrated and dehydrated groups

	Hydrated group BUN/Cre <21.4 (n = 50)	Dehydrated group BUN/Cre ≥21.4 (n = 44)	*P* value
Age, years	52.3 ± 19.0	63 ± 10.8	**0.001** ^*^
Sex, male, n (%)	33 (66.0)	15 (34.1)	**0.004** ^*^
Body mass index, kg/m^2^	24.2 ± 2.8	23.5 ± 3.0	0.216
Systolic blood pressure, mm Hg	135.7 ± 20.8	138.7 ± 19.6	0.474
Diastolic blood pressure, mm Hg	81.6 ± 13.2	80.0 ± 13.0	0.572
Habitat			
Smoking, n (%)	7 (14.0)	7 (15.9)	>0.99
Alcohol, n (%)	11 (22.0)	10 (22.7)	>0.99
Patient history			
Hypertension, n (%)	16 (32.0)	18 (40.9)	0.495
Diabetes, n (%)	5 (10.0)	8 (18.2)	0.397
Dyslipidemia, n (%)	4 (8.0)	10 (22.7)	0.087
Medical therapies			
Antihypertensive agents, n (%)	15 (30.0)	17 (38.6)	0.507
Ca blocker, n (%)	13 (26.0)	13 (29.5)	0.879
ARB, n (%)	6 (12.0)	13 (29.5)	0.063
Antidiabetic agents, n (%)	4 (8.0)	6 (13.6)	0.583
Antidyslipidemic agents, n (%)	4 (8.0)	10 (22.7)	0.087

Data is presented as medians and interquartile ranges, or means ± SDs, or ns (%s).

The ^*^(asterisk) was used to highlight a *P* value that indicates a statistically significant difference (*P* < 0.05).

ARB indicates angiotensin II receptor blocker; BUN/Cre, blood urea nitrogen-to-creatinine ratio; Ca, calcium; n, number; SD, standard deviation; SSNHL, sudden sensorineural hearing loss.

**TABLE 2.** The comparison of clinical parameters between patients with SSNHL in the hydrated and dehydrated groups

	Hydrated group BUN/Cre <21.4 (n = 50)	Dehydrated group BUN/Cre ≥21.4 (n = 44)	*P* value
Hearing parameters			
Impairment side (right), n (%)	23 (46.0)	23 (52.3)	0.689
With dizziness, n (%)	14 (28.0)	14 (31.8)	0.859
With tinnitus, n (%)	36 (72.0)	27 (61.4)	0.382
Initial hearing level, dBHL	65.0 (50.6–90.9)	80.6 (65.9–93.1)	**0.030** ^*^
Severe hearing loss (grade ≥ 3), n (%)	32 (64.0)	37 (84.1)	**0.049** ^*^
Additional treatments			
IT-DEX	13 (26.0)	12 (27.3)	>0.99
PGI2	25 (50.0)	24.5 (54.5)	0.816
Biochemical data			
Total leukocyte count, × 10^3^ cells/μL	66 (53–82)	69 (56–94)	0.319
Hemoglobin, g/dL	14.2 (13.5–15.3)	13.8 (12.7–14.6)	0.060
Platelet count, × 10^4^ cells/μL	21.0 (18.3–25.5)	22.6 (19.3–25.8)	0.304
Total cholesterol, mg/dL	206 ± 30	220 ± 29	**0.020** ^*^
Blood glucose, mg/dL	107 (98–121)	108 (102–131)	0.402
BUN, mg/dL	13.5 (11.1–15.4)	16.4 (14.3–19.9)	**<0.001** ^*^
Cre, mg/dL	0.78 (0.62–0.87)	0.62 (0.51–0.74)	**<0.001** ^*^
BUN/Cre	17.9 (16.6–20.0)	25.9 (23.4–30.2)	**<0.001** ^*^
eGFR, mL/min/1.73m^2^	80.3 ± 24.9	84.3 ± 20.0	0.405
Sodium, mEq/L	139.7 ± 2.0	140.0 ± 2.4	0.522
Potassium, mEq/L	4.3 ± 0.3	4.3 ± 0.4	0.815
Chloride, mEq/L	106 (105–108)	107 (104–108)	0.854
Plasma osmolality, mOsm/L	299.3 ± 5.0	302.0 ± 4.8	**0.010** ^*^

Data is presented as medians and interquartile ranges, or means ± SDs, or ns (%).

The ^*^(asterisk) was used to highlight a *P* value that indicates a statistically significant difference (*P* < 0.05).

BUN indicates blood urea nitrogen; BUN/Cre, blood urea nitrogen-to-creatinine ratio; Cre, creatinine; dBHL, decibel hearing level; eGFR, estimated glomerular filtration rate; IT-DEX, intratympanic dexamethasone injection; n, number; PGI2, prostaglandin I2; SD, standard deviation; SSNHL, sudden sensorineural hearing loss.

**FIG 2.**
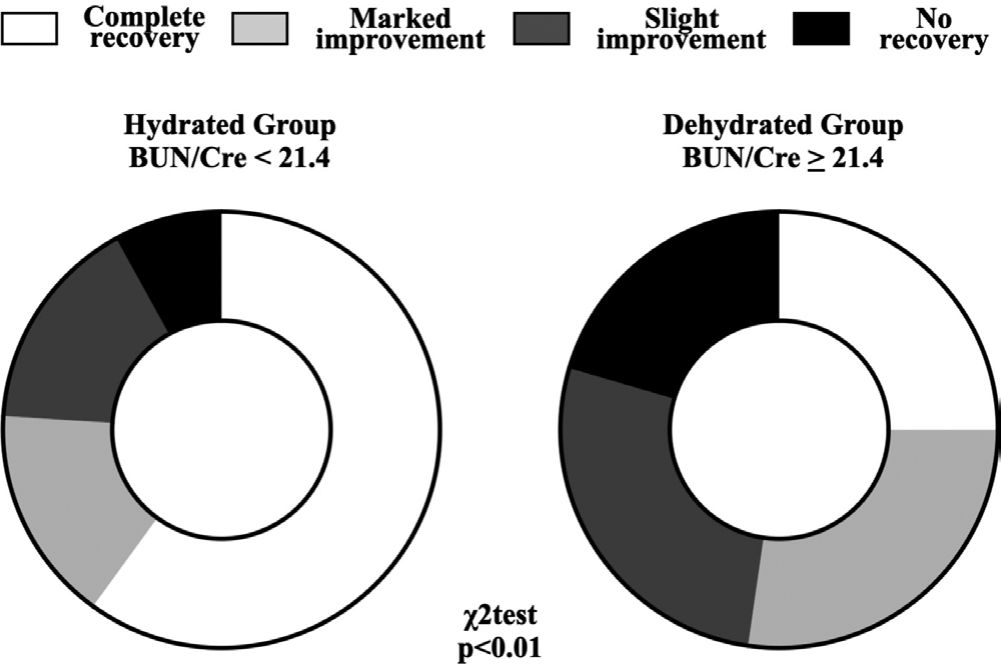
Prognosis of SSNHL in hydrated and dehydrated groups. The *χ*^2^ test shows that the recovery rate in the dehydrated group was significantly worse than in the hydrated group. BUN/Cre indicates blood urea nitrogen-to-creatinine ratio; SSNHL, sudden sensorineural hearing loss.

Figure 2 shows a comparison of the prognosis between the hydrated and dehydrated groups. The prognosis of SSNHL in the dehydrated group was significantly poorer compared with the hydrated group (*P* = 0.007). Furthermore, the residual *χ*^2^ test showed the rate of complete recovery in the dehydrated group was significantly lower than that in the hydrated group (*P* = 0.005, effect size = 0.357).

### Logistic Regression Analysis for the Prognostic Factor of SSNHL

Table 3 shows the results of the univariate logistic regression analysis for the prognostic factors of SSNHL and dehydration (BUN/Cre ≥ 21.4). Since previous reports have shown that age of more than 60 years is a worse prognostic factor for SSNHL (15,16), age was divided into 2 categories: less than 60 and 60 or more years of age. Complicating dizziness and severe hearing loss at the initial visit were associated with SSNHL’s worse prognosis, whereas age was not. Moreover, a dehydrated condition (BUN/Cre ≥21.4) was associated with a worse hearing prognosis in the univariate analysis (OR 0.22; 95% CI 0.09–0.53; *P* < 0.001).

**TABLE 3.** Univariate logistic regression of prognostic factors of SSNHL

SSNHL patients (n = 94)	Univariate logistic regression Odds ratio (95% CI)
Age, ≥60 years	0.61 (0.27–1.39)
Sex, male	1.01 (0.45–2.29)
Patient history of hypertension	0.39 (0.15–0.93)
Complicated dizziness	0.18 (0.06–0.50)
Severe hearing loss (grade ≥3)	0.32 (0.12–0.81)
Dehydration, BUN/Cre ≥ 21.4	0.22 (0.09–0.53)
Plasma osmolality, ≥299.1 mOsm/L	2.16 (0.92–5.23)
Sodium, ≥141 mEq/L	2.93 (1.26–7.04)
Total cholesterol, ≥220 mg/dL	0.81 (0.35–1.87)

95% CI indicates 95% confidence interval; BUN/Cre, blood urea nitrogen-to-creatinine ratio; dBHL, decibels hearing level; SSNHL, sudden sensorineural hearing loss.

To examine whether the BUN/Cre ratio was an independent worse prognostic factor, multivariate logistic regression analysis was performed. Three models were created for the analysis, and Table 4 shows the ORs of the 3 models. The OR of unadjusted model was 0.22 (95% CI 0.09–0.53); of model 1, adjusted for complicating dizziness, was 0.19 (95% CI 0.07–0.49); of model 2, adjusted for severe hearing loss at the initial visit, was 0.25 (95% CI 0.10–0.62); of model 3, adjusted for complicating dizziness and severe hearing loss at initial visit, was 0.22 (95% CI 0.08–0.56).

**TABLE 4.** Multivariate logistic regression of BUN/Cre and the prognostic factors of SSNHL

SSNHL patients (n = 94)	Multivariate logistic regression Odds ratio (95% CI)
Reference (unadjusted)	0.22 (0.09–0.53)
Model 1	0.19 (0.07–0.49)
Model 2	0.25 (0.10–0.62)
Model 3	0.22 (0.08–0.56)

Model 1: Adjusted for dizziness complications.

Model 2: Adjusted for severe hearing loss.

Model 3: Adjusted for complicating dizziness and severe hearing loss.

95% CI indicates confidence interval; SSNHL, sudden sensorineural hearing loss.

The restricted cubic spline showed a relationship between the BUN/Cre ratio and the worse prognosis of SSNHL (Supplemental Figure 4, http://links.lww.com/ONO/A21). This is an inverted S-shaped curve. Interestingly, the inflection point was around 20, which was widely used as a cutoff point for dehydration.

## DISCUSSION

In this study, dehydration status was significantly negatively associated with the prevalence, severity, and prognosis of SSNHL. The multivariate analysis indicated that dehydration was independently and negatively associated with SSNHL prognosis. To the best of our knowledge, this is the first study to investigate the association between hydration status and SSNHL.

In our study, the dehydration parameters, BUN/Cre and Posm, were significantly higher in patients with SSNHL than in healthy individuals. The commonly accepted reference range for BUN/Cre is under 20 (17), and for Posm is 285 ± 10 mOsm/L (18). Interestingly, the median and mean of these dehydration parameters in the patients with SSNHL were abnormal, while those in the medical checkup subjects were normal (BUN/Cre, 21.1 vs 18.8; Posm, 300.6 vs 292.2, respectively). A significantly higher number of subjects in the SSNHL group exhibited levels above the normal range for both BUN/Cre ratio and Posm when compared with the subjects in the medical checkup group (BUN/Cre ratio ≥20, 57/94 vs 40/94, *P* = 0.013; Posm ≥295, 81/94 vs 21/94, *P* < 0.0001). To demonstrate the association between dehydration and the prevalence of SSNHL, further investigations using more parameters to assess systemic water balance, such as urine volume and sodium concentration, are needed.

Patients with SSNHL were divided into dehydrated and hydrated groups based on the BUN/Cre ratio (with a cutoff point of 21.4). Interestingly, the comorbidities and habits were well-matched between the hydrated and dehydrated groups. However, the dehydrated group had a significantly higher average age and a higher ratio of women compared with the hydrated group. Previous reports have shown that BUN/Cre levels increase with age, particularly more so in females compared to males (19). Furthermore, younger men have been found to have significantly higher total body water compared with both younger and older women (20). These findings suggest that our grouping based on the BUN/Cre ratio may effectively reflect the hydration status of the patients. If we had additional parameters and data to evaluate hydration status, our findings would have even greater meaning. Further studies are warranted to evaluate hydration status using not only the BUN/Cre ratio but also physical examinations, urine analysis, and other relevant factors.

BUN/Cre ratio is useful for water balance assessments and has been reported as a prognostic factor for vascular diseases (12). Matsue et al. (21) showed that the BUN/Cre in patients with acute heart failure was higher than in the general population, and a higher BUN/Cre was independently associated with a worse prognosis. Moreover, Lin et al. (22) showed that a higher BUN/Cre ratio is a novel predictor of stroke in evolution. The results of this study showed that a higher BUN/Cre as a dehydration parameter was an independent prognostic factor for SSNHL, suggesting that hydration therapy may improve the hearing outcome of SSNHL. Several clinical reports (23,24) state that hydration therapy for stroke based on the BUN/Cr ratio may help improve prognosis and support our hypothesis. Further investigations are needed to determine whether hydration therapy for SSNHL based on the BUN/Cre ratio may improve hearing outcomes.

Systemic or intratympanic steroid therapy is widely used for the treatment of SSNHL, as steroids are known to be efficacious regarding the inner ear in cases with hearing loss of viral, vascular, autoimmune, endolymphatic hydrops, and other etiologies (25,26). Steroids also exert mineralocorticoid effects, promoting the reabsorption of sodium and water. We assumed that this mineralocorticoid activity was an additional effect of steroids on SSNHL, in addition to their protective effect on cochlear damage. In other words, systemic steroid therapy may increase blood flow to the inner ear via sodium and water reabsorption. Drugs with strong mineralocorticoid effects, such as fludrocortisone, may be the key to the treatment of SSNHL. One report showed that fludrocortisone was more effective against SSNHL than glucocorticoids and vasodilator drugs (27). However, systemic water balance was not evaluated in the previous study (27). Further studies are needed to investigate the association between drugs with strong mineralocorticoid effects and systemic water balance using several biochemical tests, such as blood and urine tests.

The renin-angiotensin-aldosterone (RAA) system is vital for regulating blood pressure, plasma electrocytes, and fluid balance (28). When blood volume or blood pressure decreases because of hemorrhage or dehydration, renin is released from the juxtaglomerular cells of the kidneys. Secreted renin activates the RAA system, increasing renal plasma flow and glomerular filtration rate (28). Yildiz et al. (29) showed that serum renin levels were higher in patients with SSNHL than in controls. One animal experiment demonstrated that renin was present in the pericytes surrounding the stria arterioles in the lateral wall of the cochlear duct (30). Thus, renin secretion may be induced by a reduction in the microvascular circulation of the stria vascularis in SSNHL to recover from vascular insufficiency and activate the RAA system. In support of our hypothesis, the estimated glomerular filtration rate in patients with SSNHL was significantly higher than that in healthy subjects (Supplement Table 2, http://links.lww.com/ONO/A23). However, although there was no statistical difference, there were more patients treated with angiotensin II receptor blockers in the dehydrated group than in the hydrated group (Table 1; *P*= 0.063).

A sensitivity analysis revealed that a higher Posm (≥299.1 mOsm/L) and a higher sodium level (≥141 mEq/L) were associated with a better prognosis (Table 3). It was hypothesized that the activated RAA system accelerated sodium reabsorption as a compensatory mechanism for dehydration recovery. In this study, plasma sodium levels carried significant weight in the estimated Posm calculation (Posm = 2 × plasma sodium + BUN/2.8 + blood glucose/18). We speculate that these factors might have contributed to the observed inconsistent result. To clarify the relationship between the RAA system and SSNHL, further in vivo, in vitro, and clinical investigations are required.

Endolymphatic hydrops (EH) have been known to be one of the reasons for acute hearing loss (31). A previous animal experiment showed that vasopressin, an antidiuretic hormone (ADH), induces EH in the inner ear of mice (32). Because ADH is secreted in response to an increase in Posm (33), dehydration may have a negative impact on EH. Interestingly, ADH estimated with Posm (0.38*[Posm-280]; pg/mL) (34) was increased in patients with SSNHL compared with medical checkup subjects in this study (7.81 [±1.91] vs 4.63 [±1.55], respectively; *P* < 0.001); therefore, patients with EH may co-exist in our cohort of SSNHL. However, recent investigations using contrast-enhanced MRI have demonstrated EH in ears with SSNHL (35), indicating that the pathology of EH partially overlaps with SSNHL. These values were estimated values and might diverge from actual measurements. Actually, these values were significantly higher when compared with the normal threshold of under 3.5 pg/mL in the previous report (36). The ADH values in our SSNHL patients were comparable to those in patients with acute-phase Meniere’s disease (37). Thus, it would be necessary to obtain accurate values by using better predictive equations based on urine osmolality or by direct measurement. Further investigation is warranted to determine the relationship between water metabolism and acute hearing loss diseases, such as SSNHL and EH, using glycerol dehydration tests or MRI testing.

The present study has several limitations. First, dehydration should be determined using multiple parameters including physical examination, urine, BUN/Cre, and plasma osmolality. Only the latter 2 were assessed in our present study. Second, the increase in BUN/Cre ratio is caused by several conditions, including dehydration, heart failure, gastrointestinal bleeding, a high protein diet, and catabolic status (17). One patient with heart failure was included in this study to maintain a sufficient number of patients for the analysis. Although the other patients with SSNHL had no comorbidity upregulating BUN/Cre and were not particular about food, their concomitant conditions were not assessed. Third, the correlation between dehydration and SSNHL was not absolute because this was a retrospective study. In our treatment protocol, blood tests were only performed upon admission. As suggested in this report, dehydration may be a potential cause of SSNHL. However, it is also worth noting that SSNHL itself may contribute to the dehydrated status. It is well known that stroke activates the sympathetic nervous system (38), which in turn stimulates the RAA system (39), potentially leading to an upregulation of the BUN/Cre ratio. If blood tests had been performed at discharge as well, the change in BUN/Cre could have been evaluated, and we could have revealed better evidence for the relationship between dehydration and SSNHL. It is therefore necessary to conduct a prospective study with hydration therapy for SSNHL. Finally, selection bias could not be ignored because this cohort study was conducted in a single center, and the population was constructed with only Japanese individuals.

In conclusion, dehydration may be associated with SSNHL development, severity, and prognosis. The dehydration parameters of BUN/Cre and Posm were higher in patients with SSNHL than in healthy subjects. Additionally, a dehydrated condition (BUN/Cre ≥21.4) was found to be an independent prognostic factor for SSNHL. However, further studies are needed to expand on this association through more thorough biochemical and physical assessments of dehydration; assessing the effects of different treatment modalities on prognoses, such as hydration, mineral corticoids, and angiotensin II receptor blockers; and analyzing the impact of other comorbidities.

## ACKNOWLEDGMENTS

We would like to thank all the staff at Jyuzen General Hospital for their excellent support during this study and Editage (www.editage.com) for English language editing.

## FUNDING SOURCES

None declared.

## CONFLICT OF INTEREST

None declared.

## DATA AVAILABILITY STATEMENT

The datasets generated during and/or analyzed during the current study are not available.

## Supplementary Material


