# Supplementary figures and images for: The Association Between Dehydration and the Prognosis of Sudden Sensorineural Hearing Loss
Source: Otol Neurotol Open. 2023 Oct 10;3(4):e041. doi: 10.1097/ONO.0000000000000041 (PMC10950149; doi:10.1097/ONO.0000000000000041)

# Supplement Figure 1

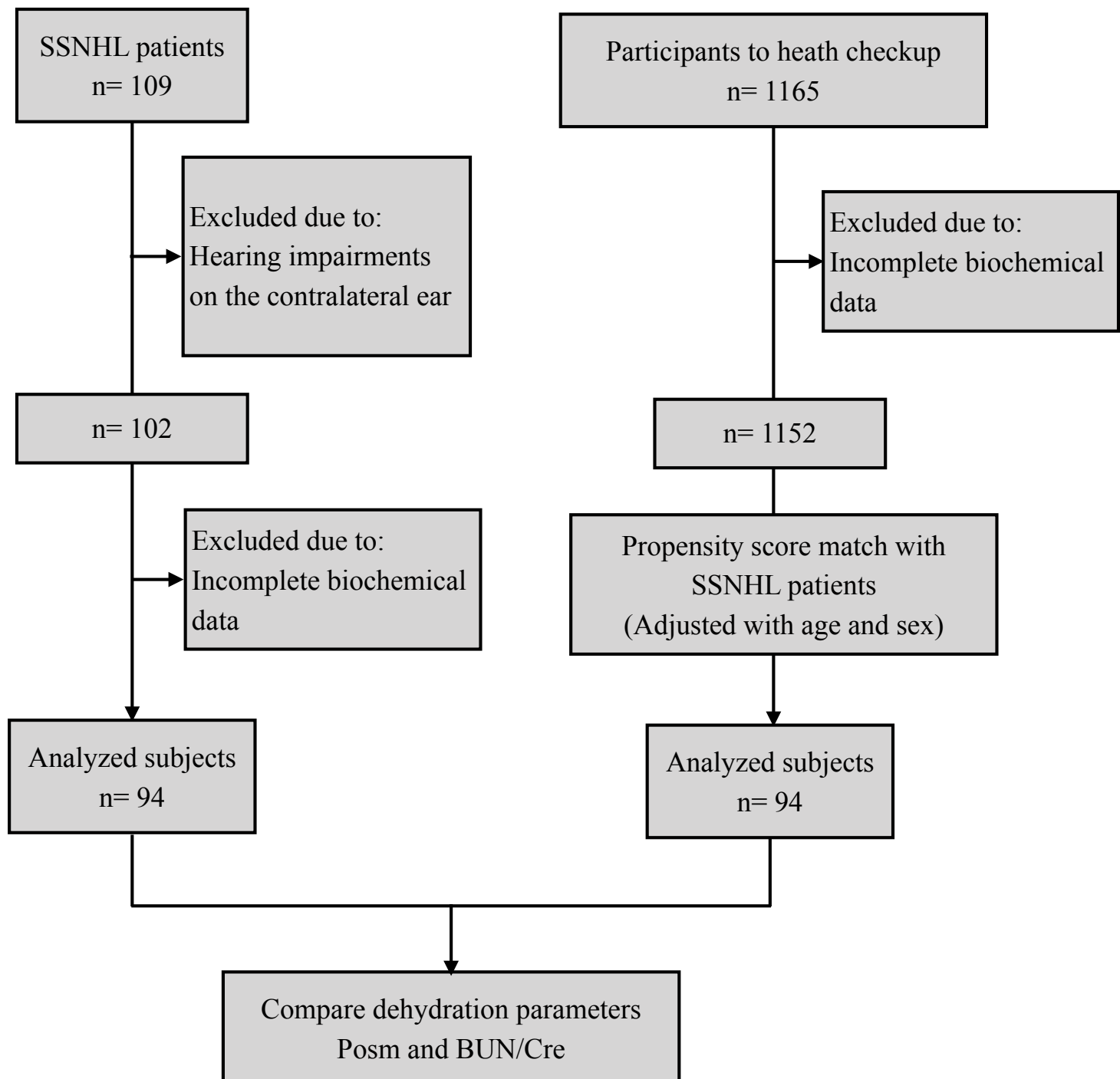

Supplement: Supplementary file 1 [file ono-3-e041-s001.pdf]

## Supplement Figure 2

(a)

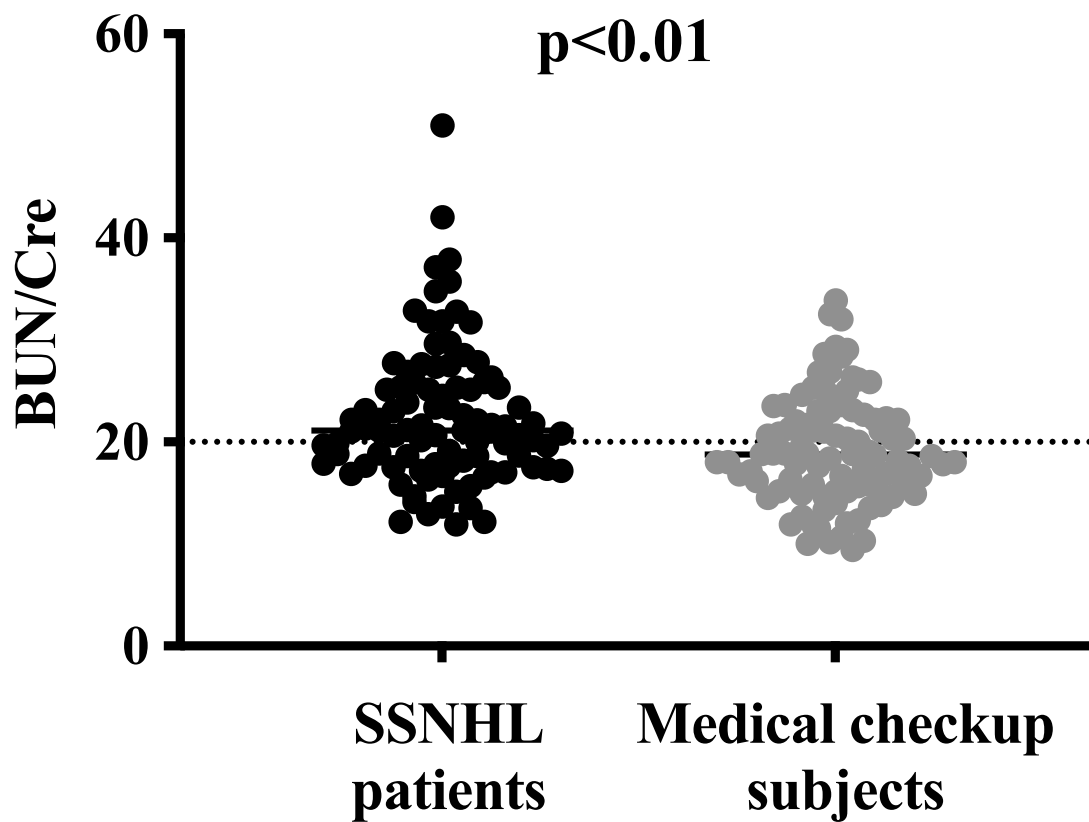

(b)

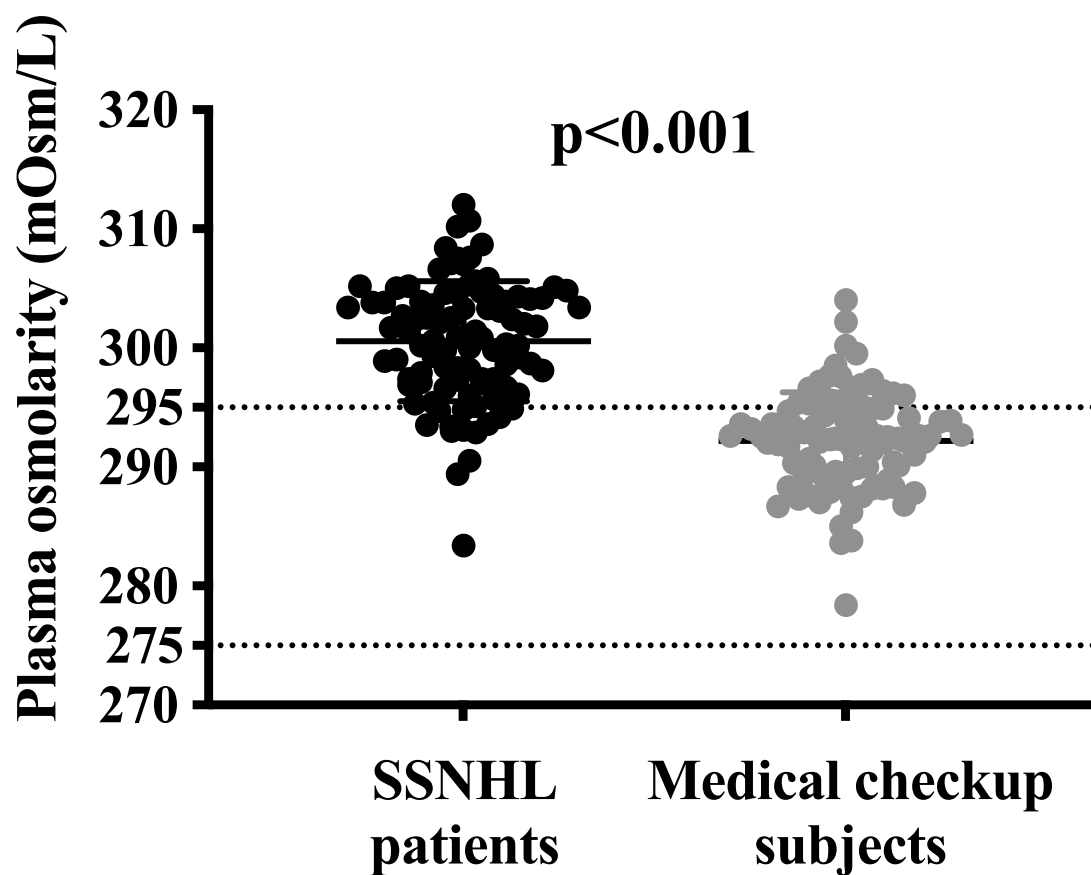

Supplement: Supplementary file 2 [file ono-3-e041-s002.pdf]

# Supplement Figure 3

(a)

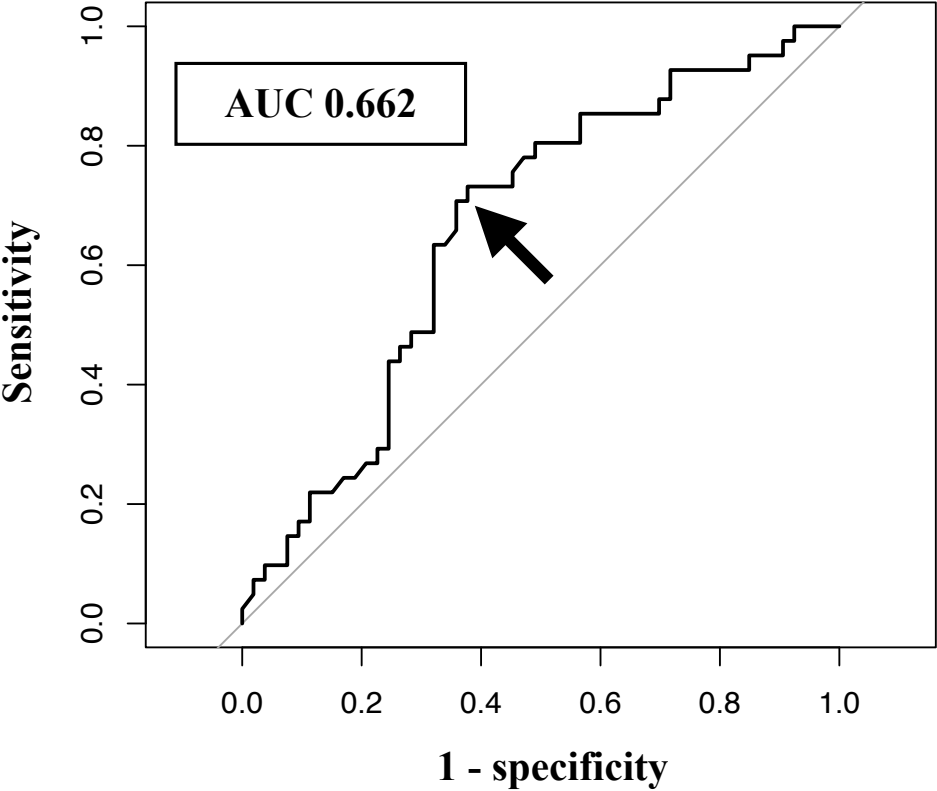

(b)

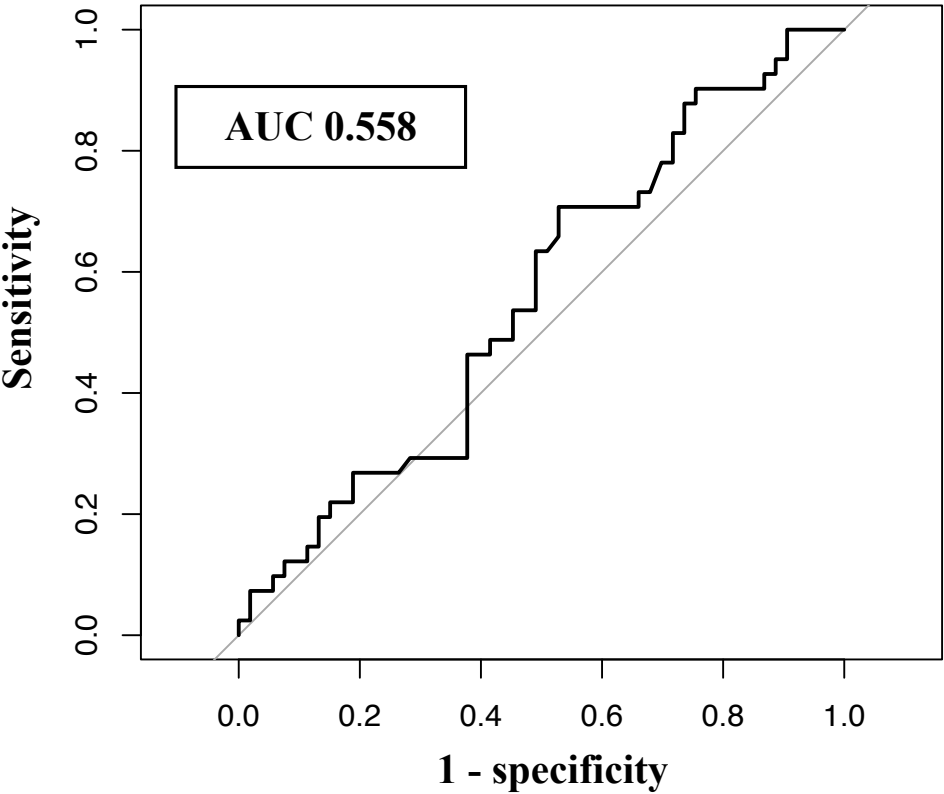

Supplement: Supplementary file 3 [file ono-3-e041-s003.pdf]

# Supplement Figure 4

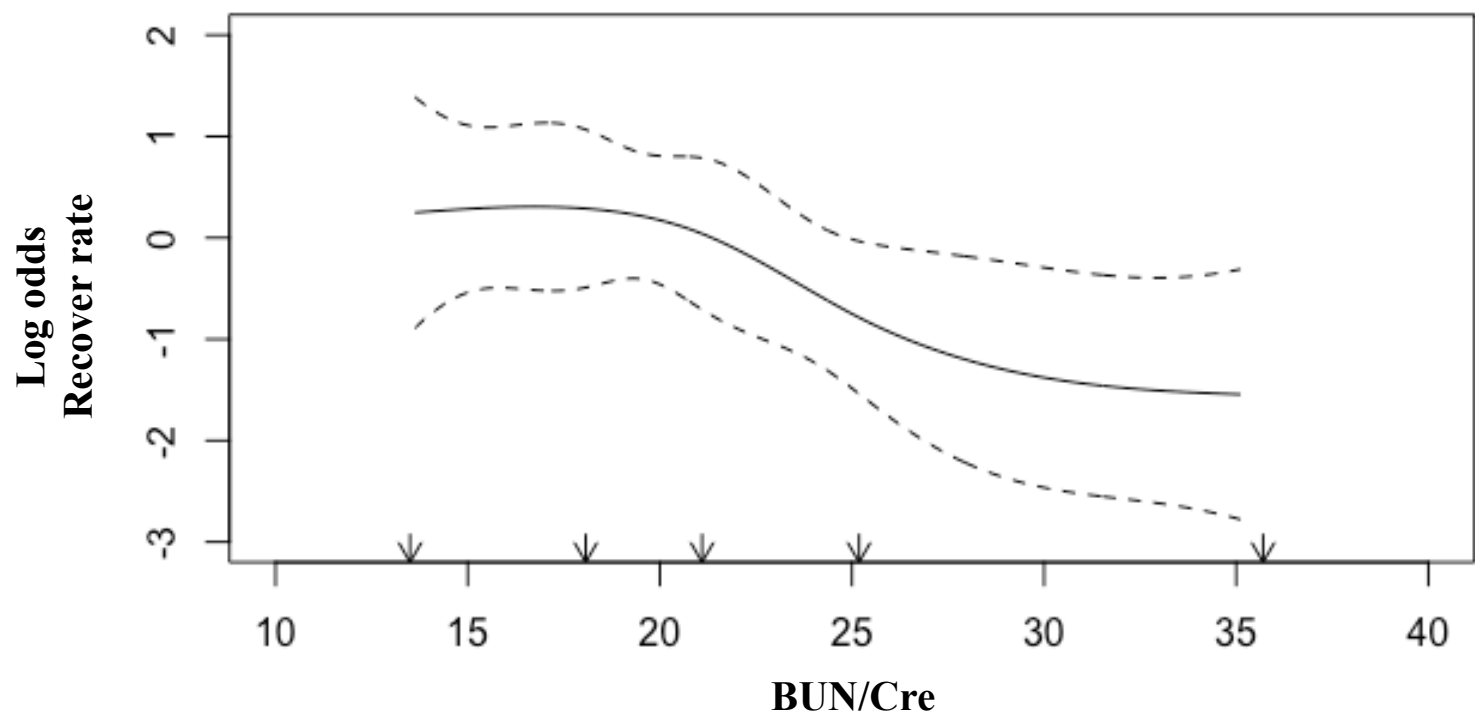

Supplement: Supplementary file 4 [file ono-3-e041-s004.pdf]
