# Supplementary material for: The Association Between Dehydration and the Prognosis of Sudden Sensorineural Hearing Loss
Source: Otol Neurotol Open. 2023 Oct 10;3(4):e041. doi: 10.1097/ONO.0000000000000041 (PMC10950149; doi:10.1097/ONO.0000000000000041)
Supplement: Supplementary file 5 [file ono-3-e041-s005.pdf]

Supplemental table 1. Characteristics of SSNHL patients and heath check-up subjects.

	SSNHL patients (n=94)	Medical check-up subjects (n=94)	*P* value
Age, years	61 (48–70)	60 (47–68)	.572
Sex, male	48 (51.1)	48 (51.1)	>.99
Body mass index, kg/m^2	23.6 (21.8–25.4)	24.5 (22.0–27.7)	.093
Systolic blood pressure, mmHg	137 (±20)	135 (±17)	.427
Diastolic blood pressure, mmHg	81 (±13)	79 (±12)	.241
Habitat			
Smoking, n (%)	14 (14.9)	13 (13.8)	>.99
Alcohol, n (%)	21 (22.3)	29 (30.9)	.248
Patient history			
Hypertension, n (%)	34 (36.2)	31 (33.0)	.759
Diabetes mellitus, n (%)	13 (13.8)	8 (8.5)	.354
Dyslipidemia, n (%)	14 (14.9)	19 (20.2)	.443
Medical therapies			
Anti-hypertensive agents, n (%)	32 (34.0)	31 (33.0)	>.99
Anti-diabetic agents, n (%)	10 (10.6)	8 (8.5)	.804
Anti-dyslipidemic agents, n (%)	14 (14.9)	19 (20.2)	.443

Data is presented as medians and interquartile ranges, or means ± SDs, or ns (%s).

Abbreviations: SSNHL, sudden sensorineural hearing loss; SD, standard deviation; n. number.
