# Supplementary material for: The Association Between Dehydration and the Prognosis of Sudden Sensorineural Hearing Loss
Source: Otol Neurotol Open. 2023 Oct 10;3(4):e041. doi: 10.1097/ONO.0000000000000041 (PMC10950149; doi:10.1097/ONO.0000000000000041)
Supplement: Supplementary file 6 [file ono-3-e041-s006.pdf]

Supplemental table 2. Clinical parameters of SSNHL patients and heath check-up subjects

	SSNHL patients (n=94)	Medical check-up subjects (n=94)	*P* value
Biochemical data			
Total leukocyte count, × 10^3 cells/μL	67 (54–87)	58 (48–68)	**<.001***
Hemoglobin, g/dL	14.0 (13.1–15.0)	14.2 (13.4–15.1)	.283
Platelet count, × 10^4 cells/μL	22.3 (18.7–25.8)	23.7 (19.6–27.1)	**.045***
Total cholesterol, mg/dL	213 (±30)	207 (±31)	.235
Blood glucose, mg/dL	107 (100–128)	104 (97–113)	**.030***
BUN, mg/dL	14.8 (11.9–17.7)	14.4 (11.4–17.0)	.125
Cre, mg/dL	0.70 (0.57–0.82)	0.76 (0.64–0.89)	**.017***
eGFR, mL/min/1.73m^2	82.2 (±22.7)	74.0 (±12.7)	**.003***
Sodium, mEq/L	140 (138–141)	141 (139–141)	**.035***
Potassium, mEq/L	4.3 (4.0–4.5)	4.3 (4.1–4.6)	.192
Chloride, mEq/L	106 (105–108)	107 (106–108)	**.039***

Data are median and interquartile range, or mean ± SD, or n (%).

Abbreviations used: SSNHL, sudden sensorineural hearing loss; BUN, blood urea nitrogen; Cre, Creatinine; eGFR, estimated glomerular filtration rate.

The * (asterisk) was used to highlight a *P* value that indicates a statistically significant difference ($P<0.05$).
